# Modeling thoracic aortic genetic variants in the zebrafish: useful for predicting clinical pathogenicity?

**DOI:** 10.3389/fcvm.2025.1480407

**Published:** 2025-02-19

**Authors:** Andrew Prendergast, Mary B. Sheppard, Jakub K. Famulski, Stefania Nicoli, Sandip Mukherjee, Patrick Sips, John A. Elefteriades

**Affiliations:** ^1^ Yale Zebrafish Research Core, Department of Comparative Medicine, Yale University School of Medicine, New Haven, CT, United States; ^2^ Department of Family and Community Medicine, Saha Aortic Center, Saha Cardiovascular Research Center, University of Kentucky College of Medicine, Lexington, KY, United States; ^3^ Department of Physiology, University of Kentucky College of Medicine, Lexington, KY, United States; ^4^ Department of Surgery, University of Kentucky College of Medicine, Lexington, KY, United States; ^5^ Department of Biology, University of Kentucky, Lexington, KY, United States; ^6^ Yale Cardiovascular Research Center, Department of Medicine (Cardiology), Yale University School of Medicine, New Haven, CT, United States; ^7^ Department of Genetics, Yale University School of Medicine, New Haven, CT, United States; ^8^ Aortic Institute at Yale-New Haven, Yale University School of Medicine, New Haven, CT, United States; ^9^ Department of Biomolecular Medicine, Ghent University, Ghent, Belgium

**Keywords:** thoracic aortic disease, zebrafish modeling, genetic variant testing, CRISPR, cardiovascular imaging

## Abstract

Thoracic aortic aneurysm and dissection (TAAD) significantly impact cardiovascular morbidity and mortality. A large subset of TAAD cases, particularly those with an earlier onset, is linked to heritable genetic defects. Despite progress in characterizing genes associated with both syndromic and non-syndromic heritable TAAD, the causative gene remains unknown in most cases. Another important bottleneck in the correct and timely diagnosis of TAAD is the large proportion of variants of unknown significance (VUS) that are routinely encountered upon medical genetic testing. Reliable functional modeling data is required to accurately identify new causal genes and to determine the pathogenicity of VUS. To address this gap, our collaborative effort—comprising teams from Yale University, University of Kentucky, and Ghent University—explores a novel approach: modeling TAAD in zebrafish. Leveraging the unique advantages of this animal model promises to allow for accelerated variant pathogenicity assessment, ultimately enhancing patient care. In this review, we critically explore the currently available zebrafish-based approaches that can be used for testing pathogenicity of genes and variants related to TAAD, and we offer an outlook on the implementation of these strategies for clinical applications.

## 1 Introduction

“Syndromic” causes of thoracic aortic aneurysm and dissection (TAAD) are defined as those associated with findings and abnormalities in both the aorta and other organ systems. Syndromic TAAD encompasses several diseases: Marfan described the earliest example of this kind of syndrome that carries his name in 1896 (although the precise genetic diagnosis for this first patient has since been questioned) (1). Ehlers-Danlos syndrome was described in 1901, and in 2005, Loeys-Dietz syndrome was described (2, 3). At the end of the 20th Century, Milewicz and colleagues and our group independently described familial inheritance of TAAD without associated syndromic features—the so-called “familial thoracic aortic aneurysm and dissection (FTAAD)” (4, 5). Since that time, 67 specific genes have become associated with thoracic aortic aneurysm to various degrees of certainty (6).

In 1999, the Human Genome Project mapped the human genome, at an estimated cost of three billion dollars (7). It took 21 more years until the first truly complete (telomere-to-telomere) sequence of the human genome was released (8). Great strides have been made toward formulation of human graph-based pangenomes (9), and databases such as gnomAD have provided an aggregated view of our existing knowledge on the standing variation in the human genome, with potential links to human diseases. Over these years, the cost of Whole Exome Sequencing (WES) [and also that of Whole Genome Sequencing (WGS)] have fallen exponentially. The current cost to run a WES assay is lower than $1,000.

The combination of a *vigorous roster of genes* associated with TAAD with the *rapidly dropping cost* of sequencing has led to an avalanche of patients who have undergone sequencing for TAAD. This is a great boon for patient, family, and science—enhancing diagnosis and enabling treatment at the *gene-specific* level. The characteristics, behavior, and severity of TAAD disease vary greatly according to the responsible gene and the specific allelic variant within that gene.

The American College of Medical Genetics and Genomics (AMG) specifies categories into which genetic variants can be classified, including (simplified for our purposes): Pathogenic, Likely Pathogenic, Variant of Unknown Significance (VUS), Likely Benign, Possibly Benign, and Benign (10). Several updates have since been proposed, such as the recent addition of the Predisposing and Likely Predisposing categories, to account for less penetrant as well as polygenic disorders (11).

The criteria for pathogenicity include the following: (1) *Frequency in the general population*. For example, TAAD is a relatively uncommon disease. If a variant is seen in greater than 1/10,000 individuals, it is too frequent to be the cause of TAAD. (2) *Preservation in phylogeny*. A gene that is preserved throughout the phylogenetic tree is likely to be very important. (3) *Effect on translational reading frame*. Frameshift mutations are likely to have a much larger functional impact than in-frame insertions or deletions as they typically lead to the inclusion of nonsense mutations in the affected transcript. This in turn can lead to nonsense-mediated decay of the entire transcript. (4) *In silico prediction*. This refers to application of computational models and large databases in analysis of specific genetic variants, which can help to predict the impact of a missense change of a specific amino acid (12). (5) *Functional data*. When the effect of a specific variant has been tested in a functional assay (either *in vitro* or, preferably, *in vivo*), this can provide strong evidence for or against pathogenicity. Despite these advanced methodologies, final confirmation of pathogenicity is ultimately determined by demonstrating *clinical correlation of genotype with phenotype over generations.* For patients with a potentially lethal condition, such as TAAD, the opportunity to evaluate how a variant affects a family through multiple generations is not always possible. Decisions for surgical threshold, medication management, and surveillance often need to be made within a matter of weeks, or less.

Furthermore, the geneticist and the surgeon often have different perspectives, of necessity. The geneticist needs to maintain scientific rigor—not wanting to designate a gene as pathogenic without strong evidence. The surgeon, on the other hand often must act based on the highest level of evidence that can be obtained within a specific timeframe. The surgeon cannot wait decades for definitive pathogenicity to be established with scientific rigor. During such an extended period, the specific patient under the surgeon's care may well succumb to a complication of TAAD, such as a fatal aortic dissection or rupture, long before scientific confirmation of pathogenicity has been accomplished.

Thus, there is a high need for a scientifically valid, but accelerated, method to determine pathogenicity for the torrent of variants identified in present day aortic care. A reliable, living organism model for rapid determination of variant pathogenicity would advance care by addressing this unmet need.

The three teams participating in this report—from Yale University, University of Kentucky, and Ghent University—have independently and concurrently embarked on projects to investigate the utility of modeling TAAD variants in a novel living organism model: the zebrafish.

The zebrafish model, were it to be shown valid, would present numerous advantages. The zebrafish is a well-established model for induction and study of genes and genetic variants. Zebrafish breed copiously, easily producing more than a hundred offspring from the mating of one couple, which can be repeated weekly. They reach sexual maturity by the age of three months, so generations can be advanced quickly and efficiently. Zebrafish are much less expensive to breed and sustain than larger models, including mice. Furthermore, zebrafish are transparent during embryonic and early larval stages, so internal organs can be visualized, even at high microscopic power, without the need for fixation, dissection, or sectioning. The availability of transgenic fluorescent reporters is a flexible, powerful asset which enables detailed *in vivo* analysis of anatomical structures [e.g., endothelial cells throughout the cardiovascular system, using the *Tg(kdrl:EGFP)* reporter (13)]. Zebrafish have already been used successfully in unbiased *in vivo* high-throughput drug screen experiments (14), owing to the scalability of experiments with embryos and larvae, and the potential for using (semi-)automated readouts of physiological effects of compound libraries. Finally, the zebrafish model is ideally suited for studies of gene function. Such functional data obtained in this animal model can provide strong evidence to classify a genetic variant according to the guidelines of the ACMG (10).

We anticipate that testing TAAD-related VUSs in zebrafish may prove to be of cardinal importance in clinical care of patients. Currently, the surgeon caring for a TAAD patient knows well how to use a report of a *disease-causing* genetic variant found on WES data. Knowing the specific causative gene carries with it specific actionable implications (15). Knowing the causative gene links the surgeon to accumulated knowledge about patients with that genetic disease (e.g., Marfan, Loeys-Dietz, Ehlers-Danlos, etc.) The surgeon knows how commonly adverse aortic events can be expected for patients with that specific heritable aortic disease, and at what aortic sizes aortic dissection and/or rupture can be expected (Supplementary Figure S1). In this way, surgical decisions become well-informed and scientifically based. In contradistinction, the surgeon does not know how to use a report of a VUS. In the VUS, the geneticist is telling us that a suspicious variant was found, but it simply is not known whether the finding is real or not. The reported VUS may be meaningful and causative of the patient's aortic disease—or it could be meaningless and not related to the patient's aortic disease. So, disease-specific clinical information about the potentially VUS related syndrome cannot logically be applied in clinical care. Patient, family, and surgeon are left uncertain and vulnerable. If zebrafish testing is shown to be accurate in delineating true pathogenicity of a VUS, patients and care givers will be dramatically empowered. It the VUS is found false, the putative findings on WES should not be applied clinically. If the VUS is found true, precise, gene-specific clinical management can be applied—often in the form of safeguarding the patient via gene-appropriate, scientifically timed surgical intervention. The present paper is intended to inform scientists and caregivers regarding the current state of the art of testing TAAD VUSs in zebrafish to clarify their true pathogenicity.

## 2 Characteristics of the zebrafish circulatory system

**Figure 1:**
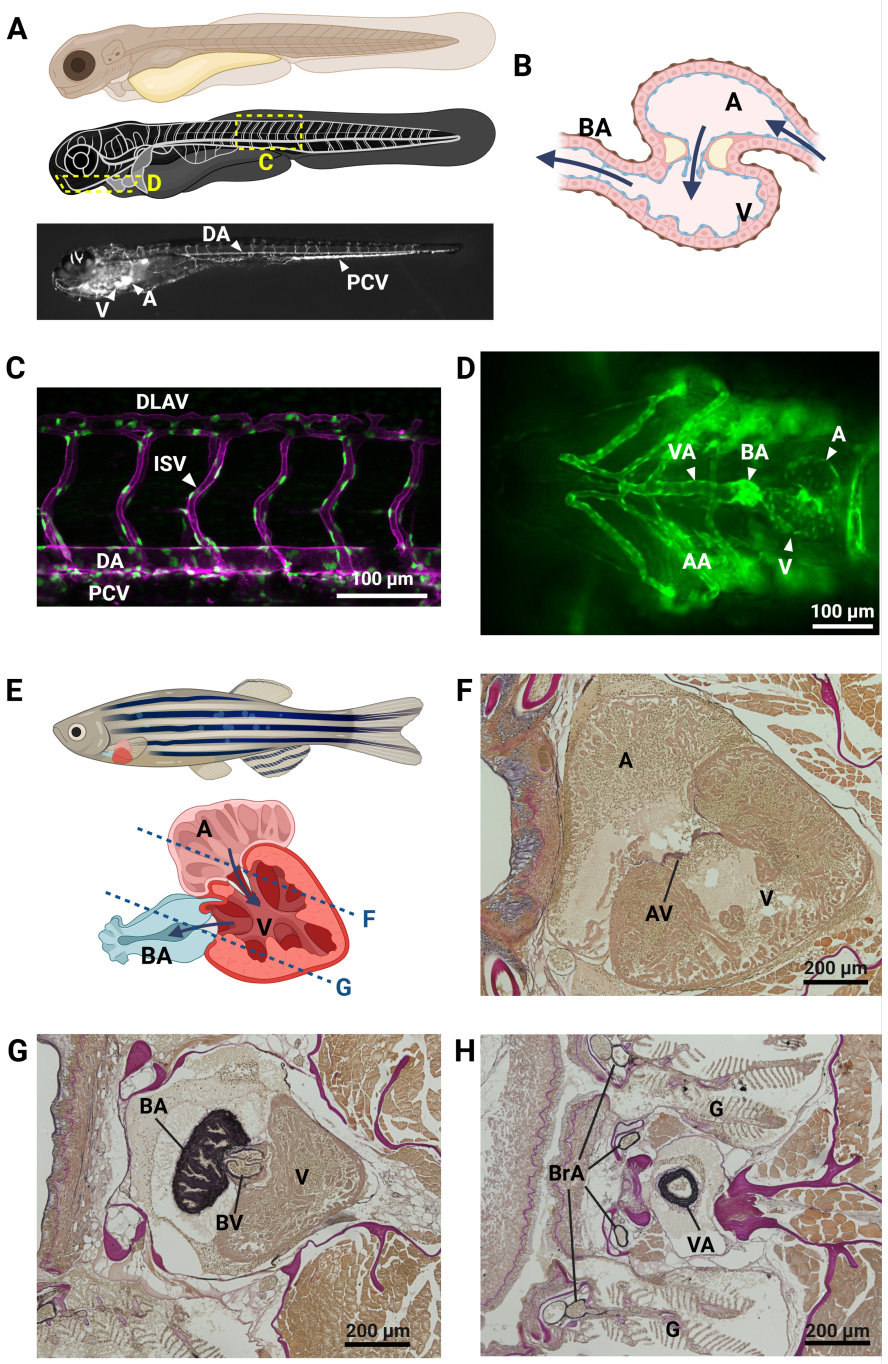
The zebrafish cardiovascular system. Representation of key elements of the zebrafish cardiovascular anatomy in embryonic/larval **(A–D)** and adult **(E–H)** stages. **(A)** Schematic overview of the circulatory system in a zebrafish embryo, showing the location of images in panels C and D. bottom: angiogram of a 5 days post fertilization (dpf) *Tg(gata1:dsRed)* zebrafish embryo, showing the trajectory of fluorescent erythrocytes throughout the circulation. **(B)** Schematic representation of the zebrafish embryo heart, with indication of blood flow direction (arrows). **(C)** Lateral view of trunk blood vessels in a 2dpf *Tg(kdrl:ras-mCherry;fli1a:nls-gfp)* zebrafish embryo, with endothelial cell membranes in magenta and endothelial cell nuclei in green. **(D)** Ventral view of the head of a *Tg(kdrl:egfp)* 4 dpf zebrafish larva, showing the heart and outflow tract. **(E)** Schematic overview of the adult zebrafish heart. The approximate planes of the cardiac sections in panels **(F)** and **(G)** are indicated with a dotted line. **(F–H)** Resorcin-fuchsin stain of elastin fibers (dark purple to black) in sections of a 9-month-old male zebrafish. **(F,G)** cardiac chambers and valves, **(H)** cross-section of the ventral aorta. AA, aortic arches; A, atrium; AV, atrioventricular valve; BA, bulbus arteriosus; BrA, branchial arteries; BV, bulboventricular valve; DA, dorsal aorta; DLAV, dorsal longitudinal anostomotic vessels; G, gills; ISV, intersegmental vessel; PCV, posterior caudal vein; V, ventricle; VA, ventral aorta. Created in BioRender. Sips, P. (2025) https://BioRender.com/z89q283.

The zebrafish circulatory system shares many features with mammalian circulation, but also retains unique characteristics (Figure 1). The zebrafish heart is a two-chambered organ which initially sends deoxygenated blood via the ventral aorta to the gill arches (Figures 1B,E–H). Blood from the ventricle is ejected via a bicuspid aortic valve into the bulbus arteriosus, an anatomic structure at the base of the ventral aorta specific to teleost fish (Figures 1B,D,E,G). The bulbus arteriosus exerts a “windkessel' effect due to its large compliance arising from its large elastin content. This function effectively dampens the pulse pressure from the ventricular outflow, thereby protecting the fragile gill capillaries from high systolic pressures (16). From an evolutionary perspective, the bulbus arteriosus is related to the aortic root and ascending aorta, which are also known to exert a “windkessel” function. Following gas exchange in the gills, oxygenated blood travels down the dorsal aorta through the entire length of the fish. At the tail of the fish, the dorsal aorta loops back to form the caudal vein, which returns blood to the heart (Figure 1A). This simple loop becomes more complex throughout phylogeny, but such is the central route of zebrafish circulation, akin to and simpler than the heart/lungs/heart/aorta/vena cava circuit in mammals.

In zebrafish studies, it is important to differentiate between zebrafish embryos/larvae and adults. Many of the advantages of zebrafish (optical transparency, small size, large sample size) are restricted to zebrafish embryos and larvae—that is, animals that are perhaps up to a week old. Unlike mammals, zebrafish develop externally and possess all major organ systems as larvae. Zebrafish move freely and feed independently by 5–6 days post fertilization (dpf). Subsequently, tissues in the zebrafish undergo profound growth and rearrangement throughout the approximately three months it takes them to reach sexual maturity. While a closed blood circulation is already functional at 1 dpf, the zebrafish cardiovascular system progressively grows and remodels with age.

In zebrafish, the first angioblasts differentiate from lateral plate mesoderm and migrate to the midline only 12 h post fertilization (hpf). These coalesce to form the arterial and venous precursors and then produce a lumen at about 26 hpf—forming the dorsal aorta and posterior cardinal vein (17). At this point, when zebrafish circulation begins, the aorta is a single-layered epithelial tube. It is critical to note that functional circulation is dispensable until about 7 days post fertilization (dpf). Thanks to the small size of the zebrafish embryo, passive diffusion is sufficient to oxygenate developing tissues (18–20). This is highly advantageous for studies of the circulatory system as severe defects that would be lethal early in mammalian development are wholly survivable in zebrafish.

Over the course of several days of development, the zebrafish aorta gradually adds vascular smooth muscle cells (vSMCs) derived from the sclerotome (ventromedial somatic mesoderm) and the aorta begins more closely to resemble the multilaminated aorta of postnatal mammals (21) (Figure 1C). This initial population of vSMCs further divides to continue the lamination of the dorsal aorta. These vSMCs are critical for the proper formation of vascular basement membrane and regulation of aortic diameter and flexibility (21). This addition of vSMCs will continue into early adulthood, at which point the aortic diameter reaches its mature size (approximately 50–70 µm). It is therefore crucial, when approaching the zebrafish as a model of human aortic disease, to consider whether disease phenomena pertaining to vSMC dysfunction are observable given the state of aortic development. The presence of other phenotypes associated with syndromic TAADs (e.g., spinal curvature, as observed in Marfan syndrome and Loeys-Dietz syndrome) may therefore also be important indicators of the pathogenicity of a variant in a particular gene.

## 3 Investigations at the contributing centers

### 3.1 The Yale University experience

In our time studying TAAD patients at Yale over nearly three decades, we have accumulated a database of 67 putative disease-causing genes (6). Studying these mutations in mammalian models would typically involve the generation of site-specific edits by homologous recombination or using CRISPR/Cas9 technology, followed by screening for germline transmission of the desired edit, an expensive and time-consuming process which would take years of work per each individual mutation. Even proceeding with germline mutants in zebrafish, with their lower housing costs and shorter generation time, such an approach would be prohibitively time-consuming and expensive.

To address these shortcomings, we have explored an F0 screen using CRISPR/Cas9 reagents. This work is dependent on multiple prior developments. Firstly, CRISPR/Cas9 ribonucleoproteins (RNPs) can be extremely effective (≥90% efficiency). Also, zebrafish are fertilized externally and can be injected with reagents at the one cell stage and immediately observed in large numbers. Thus, it is possible to generate near-totally modified embryos at scale without waiting multiple generations to isolate germline mutants. One caveat to the use of CRISPR/Cas9 is that any individual Cas9-induced double stranded break could result in an in-frame modification (presumably, 1/3 of breakage/repair events) and thereby not produce a loss of protein function. However, by injecting multiple high-efficacy RNPs per gene, it is possible to increase the overall probability of biallelic null mutations to over 90% (22). In addition, evidence suggests that recombination events at a specific CRISPR/Cas9 target site are predictable, guided by local sequence context. This information can be leveraged by appropriately trained models that can preselect gRNA sequences which will lead to a higher than random probability of a specific edit (e.g., out-of-frame mutations) (23). A possible drawback of using multiple gRNAs in a single injection is the risk of unwanted chromosomal rearrangements, which can hinder accurate interpretation of the CRISPR/Cas9 gene targeting experiment. Nevertheless, evidence suggests that, similar to local recombination events, these large-scale rearrangements are predictable, allowing appropriate *a priori* gRNA selection to minimize their occurrence (24).

It is important to note that the earliest stages of development in the zebrafish embryo are governed by maternal transcripts. The maternal-to-zygotic transition occurs predominantly before and during the initiation of gastrulation, although some maternal transcripts persist through gastrulation (25). Since an F0 CRISPR/Cas9 experiment only targets the (zygotic) genome, it is conceivable that remaining contribution of maternal transcripts and/or proteins could obscure the effects of the zygotic gene disruption, resulting in a milder or potentially even absent phenotype. An alternative strategy could therefore be to use the single strand RNA-targeting Cas13d system, which would allow targeting of maternal as well as zygotic transcripts (26). This approach has recently been used to investigate the contribution of maternal transcripts to zebrafish development (27). Nevertheless, Cas13d-induced knockdown is transient, with expression of the target gene returning after 3–5 days (26). A combined Cas13d/Cas9 approach could therefore be envisaged to achieve complete loss of the maternal and zygotic transcripts of the target gene in F0 zebrafish.

In a recently published pilot screen involving several classically known genes associated with TAAD, we scored F0 CRISPR/Cas9 fish for three major phenotypes: spinal curvature, cardiomegaly, and hemorrhage (28). In most cases, we were able to observe an increased incidence of the expected phenotypes over control levels. Furthermore, sequencing to confirm the incidence of insertion/deletion mutations found that for most genes assessed, the rate of mutation was 100% (albeit from a small number of sequenced clones). We are currently in the process of expanding this approach to a larger library of testable genes derived from patient populations.

**Figure 2:**
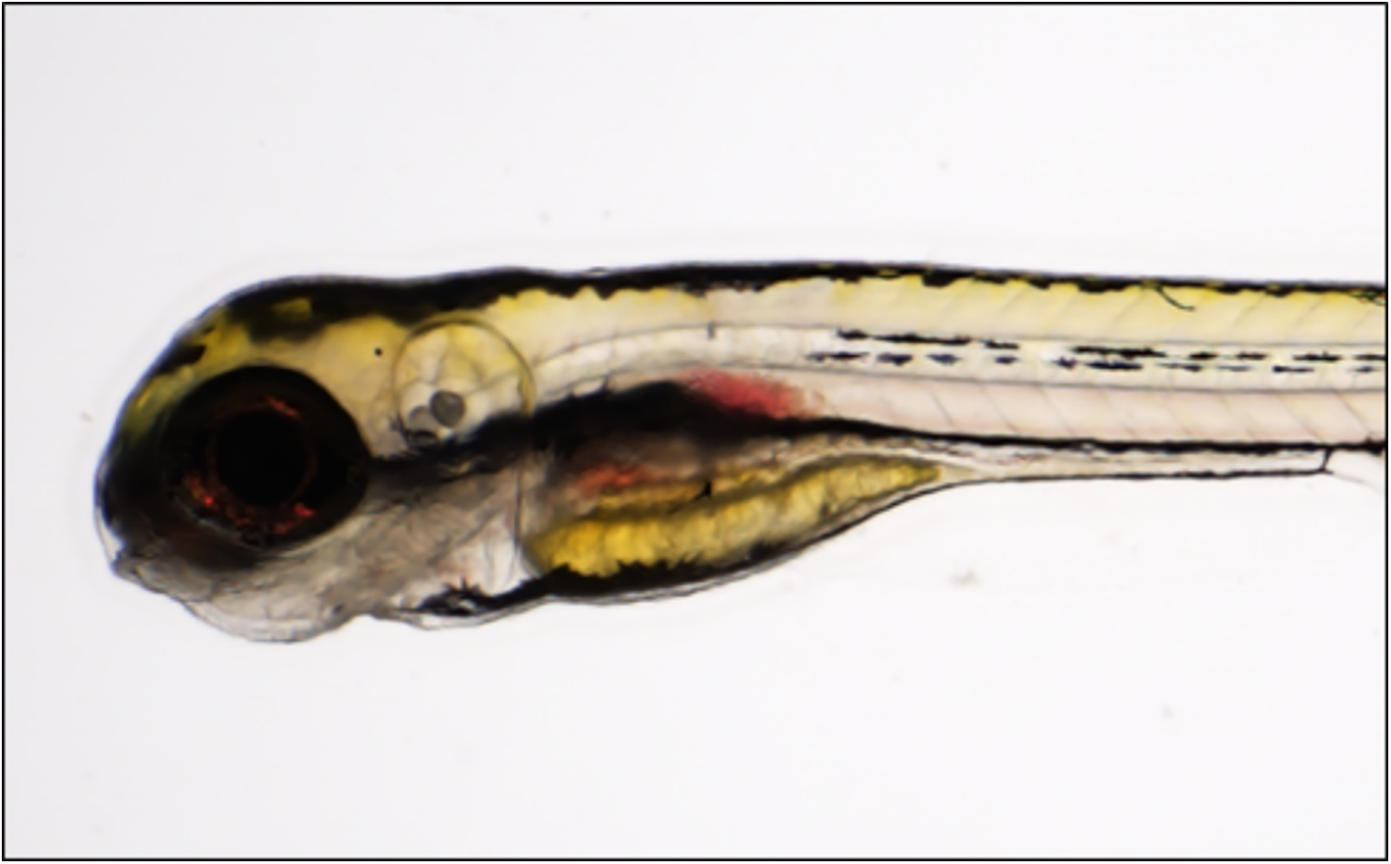
Aortic hemorrhage in a zebrafish larva. Brightfield image of a 6 days post fertilization (dpf) *col5a1*-depleted larva (CRISPR/Cas9-injected, F0 generation), showing evidence of blood pooling near the dorsal aorta (red arrow). Adapted with permission from “Knocking out Ehlers-Danlos and Marfan Syndrome-related genes generates vascular phenotypes.” by Andrew Prendergast, Bulat A. Ziganshin, Dimitra Papanikolaou, Mohammad A. Zafar, Stefania Nicoli, Sandip Mukherjee and John A. Elefteriades, licensed under CC BY 4.0.

In the 2022 study, we observed aortic hemorrhages in a small proportion of F0 CRISPR/Cas9-edited zebrafish larvae (Figure 2). The rarity of this outcome is likely due to multiple factors: first, a confluence of multiple stressors (genetic and otherwise) may be necessary to trigger aortic rupture. Second, zebrafish at early developmental stages largely lack a multi-laminated aorta and vSMCs; so, TAAD-implicated genes required for vSMC function may not be revealed by knockout experiments in very young fish. To address this, we are attempting to develop a general zebrafish model of TAAD that can be studied in older zebrafish. Mutations in human *ACTA2* are responsible for ∼15% of familial TAAD cases, making this the most prevalent single gene associated with TAAD (29). In zebrafish, we anticipate that *acta2* haploinsufficiency will generate animals sensitized to other stressors that might induce arterial dissection—as, even in humans harboring heterozygous missense mutations, hypertensive and other stressors are usually responsible for inciting the dissection event in the vulnerable aorta (30).

Preliminary work in which we have depleted *acta2* expression in zebrafish larvae using morpholino oligonucleotides increases the incidence of curvature defects and cardiomegaly as in our initial panel of TAD-associated genes (28). The use of morpholino oligonucleotides to investigate gene function nevertheless needs to be carefully scrutinized, as this technique has a number of disadvantages. Besides the transient nature of the knockdown of the target gene, artefacts due to off-target effects unfortunately are common (31). Considering the ease of generating mutants using current technologies, current guidelines recommend validating the morpholino oligonucleotide phenotype using a mutant zebrafish line if possible (32). Therefore, the true test of the *acta2* model will be in assessing the phenotype of CRISPR/Cas9-induced mutant adults. It will be very interesting to test their susceptibility to hypertensive stresses such as epinephrine administration. In a recent study of intracranial aneurysm disease (IAD) performed by our group, this treatment was capable of significantly increasing the rate of hemorrhage in a *ppil4* mutant zebrafish model of familial IAD (33). Such a model, once it is validated, might prove to be an effective platform for screening ameliorative drug therapies.

### 3.2 The Ghent University experience

Research groups at Ghent University have a long-standing interest in heritable connective tissue diseases, with a strong focus on TAAD. Basic and clinical research has been ongoing for a range of genetic disorders, including cutis laxa, Ehlers-Danlos syndrome, Loeys-Dietz syndrome, and Marfan syndrome. Different zebrafish models have been generated at Ghent University for these disorders, with the aim of producing new tools that can advance our understanding of basic disease mechanisms, enable identification of new treatment options, and test the pathogenicity of novel genes and variants. Using CRISPR/Cas9 technology, stable mutant lines have already been generated, several of which have been published recently (34–38). In general, these zebrafish models recapitulate characteristics relevant to the human disease, confirming their role as a relevant model. Cardiovascular phenotypes have already been documented in these mutant zebrafish. Validation of zebrafish as a suitable animal model for studying thoracic aortic disease is essential before this model can be considered for variant testing. As a first example, loss of *atp6v1e1b*, the ortholog of the human *ATP6V1E1* gene that is associated with autosomal recessive cutis laxa type 2C, leads to aberrant development of aortic arches and proximal ventral aorta in zebrafish, which could be correlated to the thoracic aortic disease observed in cutis laxa patients (38).

Ongoing work is focused on the study of zebrafish orthologs of different fibrillin and *SMAD* genes to investigate their impact on aortic homeostasis, considering their role in Marfan syndrome, bicuspid aortic valve, and Loeys-Dietz syndrome in humans. The results of these studies will be described in detail in forthcoming publications. For the phenotype of the fibrillin mutant zebrafish, particular focus is on the structure of the bulbus arteriosus, as this anatomical structure has an evolutionary link with the human aortic root, which is typically dilated in patients with Marfan syndrome. A strong endocardial phenotype has previously been described in *fbn2b* mutant zebrafish embryos, indicating that fibrillin function is also important in early cardiac development (39). To be able to study the function of *SMAD3* and *SMAD6* in zebrafish, both the *smad3a* and *smad3b* as well as the *smad6a* and *smad6b* ohnologs, which are the result of the whole genome duplication which occurred in the common ancestor of the teleost fish lineage (40), needed to be targeted due to their respective redundancy. Strikingly, *smad3a/b;smad6a/b* quadruple mutant zebrafish demonstrate a ventral aortic phenotype in adult stages which strongly mimics human aortic dissection (41). These data confirm that reproducible phenotypes relevant to human TAAD can be induced in zebrafish by genetically targeting functional orthologs of human disease-causing genes.

Past and current zebrafish studies have mostly taken advantage of the high efficacy to obtain insertion and deletions resulting from non-homologous end joining (NHEJ) repair after a CRISPR/Cas9-induced double-stranded break in a gene of interest, leading to the generation of loss-of-function alleles. Such studies can be very informative with regard to the potential pathogenic role of a specific genetic deficiency. Nevertheless, to enable accurate modeling of the majority of VUS encountered in medical genetics, it is important to be able to investigate missense mutations in zebrafish genes of interest as well. Previous work has shown that homology-directed repair (HDR) can be used to introduce specific targeted edits into the zebrafish genome by co-administrating a repair template together with the CRISPR/Cas9 complex. Nevertheless, the efficiency remains very low (range of 1%–5%), despite multiple efforts by our group and others in testing different modifications of the HDR protocol, including different lengths and modifications of the repair template (42, 43). Since NHEJ repair is more efficient than HDR in these experiments (leading to more indels than correct repair at the desired genomic position), it is not feasible to monitor effects of specific genome edits in the F0 generation. Therefore, such studies require selection of appropriate founders and subsequent establishment of a stable line carrying the desired modified allele. New CRISPR prime editing technology holds the promise of significantly boosting the efficiency of targeted genome editing (potentially up to ∼30%) (44, 45). Ongoing experiments in our center are now focused on optimizing prime editing technology for use in zebrafish models, which might lead to higher efficacy in combination with lower off-target effects.

Most research efforts have been devoted to observing relevant phenotypes in early stages of zebrafish development. This opens up the possibility of a fast turnaround time for studying potential pathogenicity of new candidate genes and/or variants and enables high-throughput chemical compound screening to find new modulators of aortic disease. Nevertheless, after generating stable mutant lines, it is also possible to evaluate progressive phenotypes that only develop at a later age. As highlighted in the previous section this approach is likely inevitable when the effects of specific missense mutations or other complex genotypes need to be studied. While this will lower the throughput of the number of genes/variants that can be tested, it can still provide essential information on pathogenesis—as was the case for the quadruple *smad3a/b;smad6a/b* knockout zebrafish line that we have been studying in our center. Considering the efficacy of generating genetically modified zebrafish lines, this is still more cost-efficient than generating the respective mouse mutants, especially when also taking into account the species-specific husbandry advantages, as well as the flexibility of studying large numbers of offspring in subsequent generations.

We have recently developed and optimized a toolkit for systematic and reproducible analysis of adult zebrafish cardiovascular phenotypes. Since zebrafish lose their optical transparency during larval development, the only way to assess adult cardiovascular function *in vivo* is via ultrasound analysis. Based on prior studies (46, 47), we have optimized a standard protocol to reliably perform B-mode as well as pulsed-wave Doppler measurements of the adult zebrafish heart. We also implemented an automatic workflow to efficiently analyze zebrafish pulsed-wave Doppler recordings, and used this to generate reference data based on a large set of wild type measurements (48).

We have also successfully used synchrotron-based phase-contrast propagated micro-computed tomography to image the soft tissue structure in fixed adult zebrafish. From these images, the structure of the heart and main blood vessels can be segmented in a relatively straightforward way. The resulting three-dimensional reconstructions can then be used to visualize structural abnormalities and/or compute biomechanical models of zebrafish blood circulation (49). To obtain a better representation of vascular morphology *in vivo*, we adapted existing vascular corrosion casting protocols for use in zebrafish, allowing the fixation of cardiovascular structures at a maintained intraluminal pressure via injection of a resin that polymerizes *in situ* (50). Recently, we have characterized a Hafnium-based contrast agent which can be added to the injected resin, obviating the need for the corrosion of soft tissue before visualization of the vascular tree using micro-computed tomography (51).

### 3.3 The University of Kentucky experience

Investigations at the University of Kentucky have focused upon the injection of mRNA mimicking human variants of uncertain significance in the *smad3a* gene during the 1-cell embryonic stage of development in zebrafish. Characterization of the dorsal aorta at 48 hpf was used to assess phenotypes. In a proof of principle study, the Kentucky team sought out to determine whether the zebrafish embryo assay could provide *in vivo* assessment of a novel *SMAD3* VUS. This serves as one of the first known cases of a zebrafish modeling experiment which provided important information that was used for clinical decision making in a patient with heritable aortic disease.

#### 3.3.1 Case study

*Details of this clinical case have been published recently* (52)*. In brief, a 55-year-old male with a 4.5 cm aortic root aneurysm, paroxysmal atrial fibrillation, hypertension, and eosinophilic esophagitis had presented to the University of Kentucky Aortic Clinic for evaluation of heritable thoracic aortic disease. The patient had a positive family history for abdominal aortic aneurysm, atrial fibrillation, and aortic dissection. Invitae aortopathy panel was negative for pathogenic variants, but identified a heterozygous variant of uncertain significance in SMAD3, c730G>T (p.Val244Phe). The patient's parents and brother were not willing to undergo segregation analysis. Segregation analysis in the patient's three children was deferred given their young age, given the risk of a clinical false negative (i.e., they may not have had time to fully develop the aortopathy phenotype, even if the variant was present and disease-causing)*.

Multiple clinical decisions hinged upon the definitive classification of the variant as pathogenic. Most importantly, if the variant was pathogenic, the patient's aortic diameter of 4.5 cm was at the surgical threshold to undergo valve-sparing aortic root replacement for individuals with *SMAD3* mutations (53). In the absence of a pathogenic variant, the surgical threshold for repair was 5.5 cm. Second, the presence of a pathogenic *SMAD3* variant could alter pharmacologic treatment options. Third, if the variant was pathogenic, additional vascular screening would be recommended, including a magnetic resonance angiography of the head, neck, abdomen, and pelvis.

Given the need to accrue as much data as possible and the absence of time or resources to model the variant in mouse, the zebrafish embryo system was used to investigate potential pathogenicity. Injection of mRNA is routine in the field of zebrafish developmental biology, but in the case of FTAAD, it offered a unique opportunity (52, 54). Knowing that variants of *SMAD3* have been shown to have dominant effects on pathology, coupled with the very high degree of *SMAD3* conservation in zebrafish, we investigated whether injection of *SMAD3* VUS mRNA could function as a test of potential pathogenicity, using integrity of the newly formed zebrafish vasculature as a readout. The approach involved injection of mRNA harboring the V244P mutation in the zebrafish *smad3a* gene, which has over 90% homology with human *SMAD3*. Using a known pathogenic variant (T2611) as control, we were able to show that injection of novel *SMAD3* VUS V244F mRNA results in slight, but significant, enlargement of the dorsal aorta in the zebrafish embryo, as visualized using the transgenic *Tg[kdrl:mCherry]* zebrafish line where vasculature endothelial cells are labelled by mCherry fluorescence (55). We then expanded our investigation by examining additional *SMAD3* VUSs. Analysis of six previously uncharacterized VUSs revealed that P124T, L296P and A349P were also potentially pathogenic according to their zebrafish embryo assay (52). Overall, our study was able to showcase the potential of using zebrafish embryos as an *in vivo* readout for molecular function, and therefore, assay for potential pathogenicity of *SMAD3* VUSs.

The fact that these studies were able to be conducted using zebrafish embryos offers the potential for truly personalized medicine based on VUS identification as well as offering a potential screening for treatment options. In an ideal example, it could take as little as two weeks from the time a patient's sequencing results identify a VUS, to mRNA synthesis, injections, and ultimately analysis of vascular phenotype in 48 hpf embryos. The observed results could then be used to inform treatment options, as regarding the patient harboring the novel V244F *SMAD3* variant. At the time of variant diagnosis, the patient elected to defer valve-sparing aortic root replacement. Nevertheless, as the patient was already taking a beta-blocker as anti-hypertensive medication, he agreed to change his regimen to include an angiotensin-receptor blocker (ARB). This treatment would not have been considered if we did not have the information regarding the pathogenicity of the V244F *SMAD3* variant based on our zebrafish study.

In many clinical circumstances, the level of certainty provided by this type of zebrafish-based assay may not be sufficient to change decision-making. However, this approach offers a high-throughput assay at low cost that can then be validated using other techniques. For example, promising therapeutics can be screened in zebrafish and then taken to preclinical murine studies, which often take years to conduct and may cost more than $100,000 to investigate a single therapeutic. In times of clinical decision making where an individual may have only weeks or months to decide, but not years (as described in the case above), a zebrafish assay may provide valuable information, which if accrued with appropriate controls and scientific rigor, is better than no information at all.

## 4 Work at other centers

Work has been done by other groups (beyond the three reporting in this paper) utilizing the zebrafish model in TAAD. Several groups have modeled genetic deficiencies associated with TAAD in patients. Modeling of *SECISBP2* deficiency, which in patients was associated with TAAD, resulted in dilation of the ventral aorta in zebrafish larvae which was significantly exacerbated by exposure to oxidative stress. These results confirmed the hypothesis that deficiency of antioxidant selenocysteine-containing proteins resulting from the loss of *SECISBP2* function leads to oxidative stress-induced aortopathy (56). In another study, a zebrafish model was used to test the effects of a human *ACTA2* variant identified in a patient with TAAD, acute Stanford type B dissection and left ventricular non-compaction. They used an approach similar to the study of the Kentucky group, injecting zebrafish embryos with mRNA encoding the human *ACTA2* variant. They did not report any aortic phenotype in the zebrafish larvae however, but they did confirm a cardiac non-compaction phenotype concordant with the human case (57). An older study tested the effects of two variants of the *MAT2A* gene, which predispose to TAAD in patients, in zebrafish larvae in which the endogenous *mat2aa* gene was knocked down using morpholino injection. *mat2aa* knockdown resulted in developmental defects including malformation of the aortic arches. This morpholino-induced phenotype was rescued to a larger extent by coinjection of wild-type *MAT2A* mRNA than by mRNA encoding a *MAT2A* variant (58). A subsequent publication from this research group used the same approach to show that mRNA encoding a human variant in *FOXE3*, linked to thoracic aortic dissection, could not rescue the defect in aortic arches development observed in zebrafish larvae injected with *foxe3*-targeted morpholinos (59). Modeling of a hypomorphic allele of *robo4*, mimicking variants associated with TAAD and bicuspid aortic valve in patients, resulted in regurgitation from the ventriculo-bulbar valve in adult zebrafish, although no aortic phenotype was detected (60).

In work by other groups, the zebrafish model has been utilized predominantly to elucidate the mechanisms involved in aortic homeostasis, with a focus on the role of transforming growth factor-beta (TGF-β) signaling. Abrial and colleagues found that compound loss of both latent TGF-β binding proteins 1 and 3 resulted in severe aortic outflow tract dilatation in zebrafish larvae, accompanied by ventricular dilatation. This effect could be mimicked by pharmacological inhibition of the TGF-β type I receptor, demonstrating that TGF-β signaling protects against developing aneurysmal disease (61). These results confirm a prior study showing that loss of the TGF-β type I receptor leads to striking outflow tract dilatation and loss of ventral aorta development in early zebrafish larvae. This phenotype could be rescued by endothelial-specific overexpression of the TGF-β type I receptor, demonstrating the importance of this cell type for early aortic development (62). Earlier work demonstrated that morpholino-based knock-down of *SKI*, a known repressor of TGF-β signaling associated with Shprintzen-Goldberg syndrome, resulted in malformations in the outflow tract of early zebrafish larvae, although a complete description of the phenotype was lacking (63).

Taken together, this important work by multiple teams underscores the increasing support for zebrafish as a model for TAAD, and has already helped to increase our understanding of the mechanisms involved in aortic homeostasis which are disrupted in TAAD. This research paves the way for successful broad implementation of the zebrafish model for the assessment of clinical significance of specific genetic variants.

## 5 Discussion

**Figure 3:**
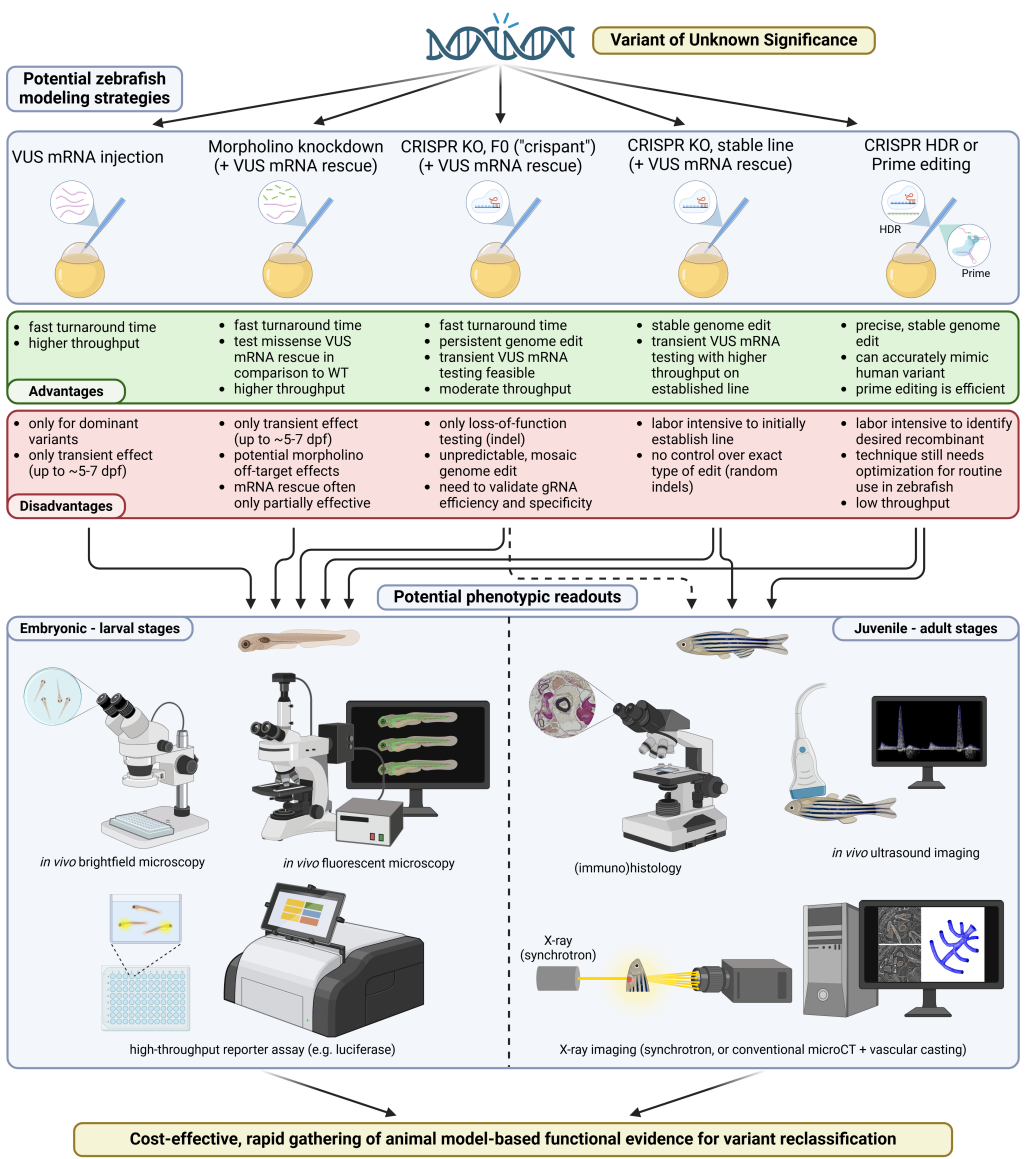
Overview of zebrafish strategies for the modeling of variants in genes associated with TAAD. Several different strategies are currently available for gene and variant testing in zebrafish. Each approach has specific advantages and disadvantages, rendering it more or less suitable for various purposes. Depending on the chosen strategy, phenotyping of the zebrafish can be performed in embryonic and larval stages, or also at later ages. Created in BioRender. Sips, P. (2025) https://BioRender.com/z58k940.

As seen in this comprehensive review, experience modeling thoracic aortic diseases in the zebrafish is advancing. Figure 3 summarizes a number of approaches that can be used for VUS modeling in the zebrafish model, listing their respective advantages and disadvantages. Supplementary Table S1 recapitulates the TAAD genes that have already been tested in the zebrafish. A zebrafish phenotype for TAAD has indeed emerged, that appears substantially to recapitulate the disease in humans. Modeling has been predominantly limited to embryonic stages of zebrafish development, although the Ghent University group has amassed substantial experience in later, adult stages. Where do we go from here? We queried each center individually for their thought and plans.

Specifically, we posed to each of our three groups the following question:

Do you feel that assessment of TAAD Variants of Unknown Significance (VUSs) in the zebrafish model is ready for clinical application, albeit with clearly emphasized provisos and disclaimers? Yes, No, or maybe, and why?

***Yale University Group***: Of course, we subscribe to extreme rigor in assessment of the genetic significance of detected irregularities in genes associated with TAAD. Infrequency of a variant in the general population, preservation in phylogeny, and various in silico parameters serve to inform this assessment. However, we do feel that the VUS designation may leave clinicians, patients, and families in an uncomfortable quandary. We are especially sensitive to this as, although a multidisciplinary team (genetics, cardiology, cardiac surgery), we are actively involved and responsible for the care of large volumes of individual TAAD patients. Patient decisions may not be able to wait for ultimate, unequivocal, scientifically impeccable designation of the pathogenicity of a specific variant in an individual patient. Certainly, waiting to confirm genetic significance by segregation of phenotype with genotype, which may take generations, is beyond the safety window for specific patients. In clinical practice, we generally tend to “believe” VUSs in patients with plausible variants and concerning ascending aneurysms. We often treat surgically—to assure patient safety from rupture—and follow accrual of specific variant data long-term post-operatively. Such a surgical decision is encouraged by the rapidly advancing safety of ascending aortic surgery in the present era (∼98.5% survival). With this surgical perspective in mind, we feel that evaluation of TAAD variants in zebrafish stands at the very threshold of clinical applicability. We are currently evaluating zebrafish discriminatory accuracy by challenging our zebrafish model with two batteries of variants—one known pathogenic and one known benign. Early findings suggest accurate discrimination based on zebrafish phenotype after induction of the variant. Furthermore, we wish to follow our zebrafish into later F2 and F3 generations. These colonies are currently reaching maturity and should permit recognition of additional phenotypic features.

Bottom line: we find ourselves right on the cusp of accepting zebrafish testing of TAAD variants as substantially informative.

***Ghent University Group***: Considerable progress has been made recently in the field of zebrafish modelling of TAAD, with technological capabilities advancing to the point where experiments in zebrafish can be used to assist with the assessment and potential re-classification of VUS. Nevertheless, some important caveats must be considered. While loss-of-function alleles can be addressed in a relatively straightforward manner using current genome editing technologies, the modeling of specific missense variants still carries some limitations due to the unpredictable efficacy of mRNA complementation experiments. More efficient genome editing tools are constantly being developed and will likely alleviate this concern in the near future. Importantly, zebrafish testing modalities need to be adequately benchmarked against known pathogenic and benign variants to be able to make confident predictions regarding pathogenicity, in line with ClinGen recommendations (64).

Another important consideration is that a large proportion of human genes have multiple orthologs in the zebrafish genome due to the teleost whole genome duplication (65). Since many of the zebrafish ohnologues have redundant functionality, they would need to both be targeted to elicit the desired phenotype in zebrafish, as we experienced for the *smad3a/b* and *smad6a/b* genes. Finally, relevant aortic phenotypes may be progressive and only become detectable at later (adult) stages. While this prolongs the turnaround time and decreases throughput for a zebrafish assay of variant pathogenicity, it could still provide clinically useful information. Advances in genome editing technology resulting in increased recombination efficacy will enable screening in F0 zebrafish, precluding the need to wait for multiple generations of fish to grow up.

Bottom line: new technological developments have brought the implementation of the zebrafish model for variant testing within reach, but more validation is needed before zebrafish data can be applied for routine clinical decision-making in TAAD.

***University of Kentucky Group***: The zebrafish embryo model has definite limitations that must be considered when considering clinical applications. One is the lack of a clear regional-specific correlation between the zebrafish aorta and regionally-specific demarcation of an ascending thoracic, descending thoracic, and abdominal aorta in humans. For some models, this may be relevant. For *SMAD3*, this was not considered to be invalidating because humans with *SMAD3* pathogenic variants are known to have aortic aneurysms throughout all three regions of the aorta. Second, a variant mRNA injection approach is limited to modeling disease at early stages of development, due to the limited half-life of the injected mRNA. To model disease at later stages of aortic development, a germline mutation would need to be created, which using current technology considerably increases study time and expense. Third, the mRNA injection approach is most useful for specific types of variants. Nonsense variants and variants that cause a frameshift in mRNA translation usually are not dominant, so they are not amenable to this approach. The example of *SMAD3* VUSs relied on the well-known fact that *SMAD3* mutations can have dominant effects. Loss of function VUSs, often being frameshift insertions or deletions, would not be an option for testing in this system due to endogenous wildtype protein expression. Importantly though, there are several other FTAAD related genes that exhibit dominant effects and would therefore be suitable for testing in this fashion.

Since not all physiological aspects of FTAAD will be represented easily in early zebrafish development, such as the examination of vasculature at 48 hpf, additional tools and approaches are needed. Unfortunately, mRNA injections become too depleted after 48 hpf to be reliable for older stage studies. However, zebrafish offer a plethora of genetic options, including induction of the VUS cDNA using heat shock promoters, tissue specific promoters, or knock-in experiments that would model patient mutations. Furthermore, libraries of known pathogenic mRNA would enable large scale drug screening for therapies for which zebrafish are known to be susceptible, providing a high throughput vertebrate model.

Bottom line: in carefully selected cases and when testing genes that have well-validated and reproducible phenotypes in the zebrafish, it could be reasonable to use zebrafish for assessment of TAAD VUS. Unfortunately, we do not currently have a well-validated and reproducible phenotype in the zebrafish for the majority of TAAD genes. This critical work still needs to be done to establish a sufficient groundwork for clinical applications. We feel that this barrier can be overcome with dedicated resources and collaboration among scientists.

## 6 Conclusions

Three zebrafish laboratories—Ghent, Kentucky, and Yale–have independently pursued testing of VUSs in TAAD disease. Their programs and early findings are presented and evaluated herein. It is clear already that the zebrafish may well prove extremely useful in clinical designation of pathogenicity of specific novel VUS in TAAD disease. This may well enhance surgical decision making in the near future.
