# Supplementary material for: Modeling thoracic aortic genetic variants in the zebrafish: useful for predicting clinical pathogenicity?
Source: Front Cardiovasc Med. 2025 Feb 19;12:1480407. doi: 10.3389/fcvm.2025.1480407 (PMC11892108; doi:10.3389/fcvm.2025.1480407)
Supplement: Supplementary file 1 [file Datasheet1.docx]

Supplementary Material

# Supplementary Figures and Tables

## Supplementary Figures


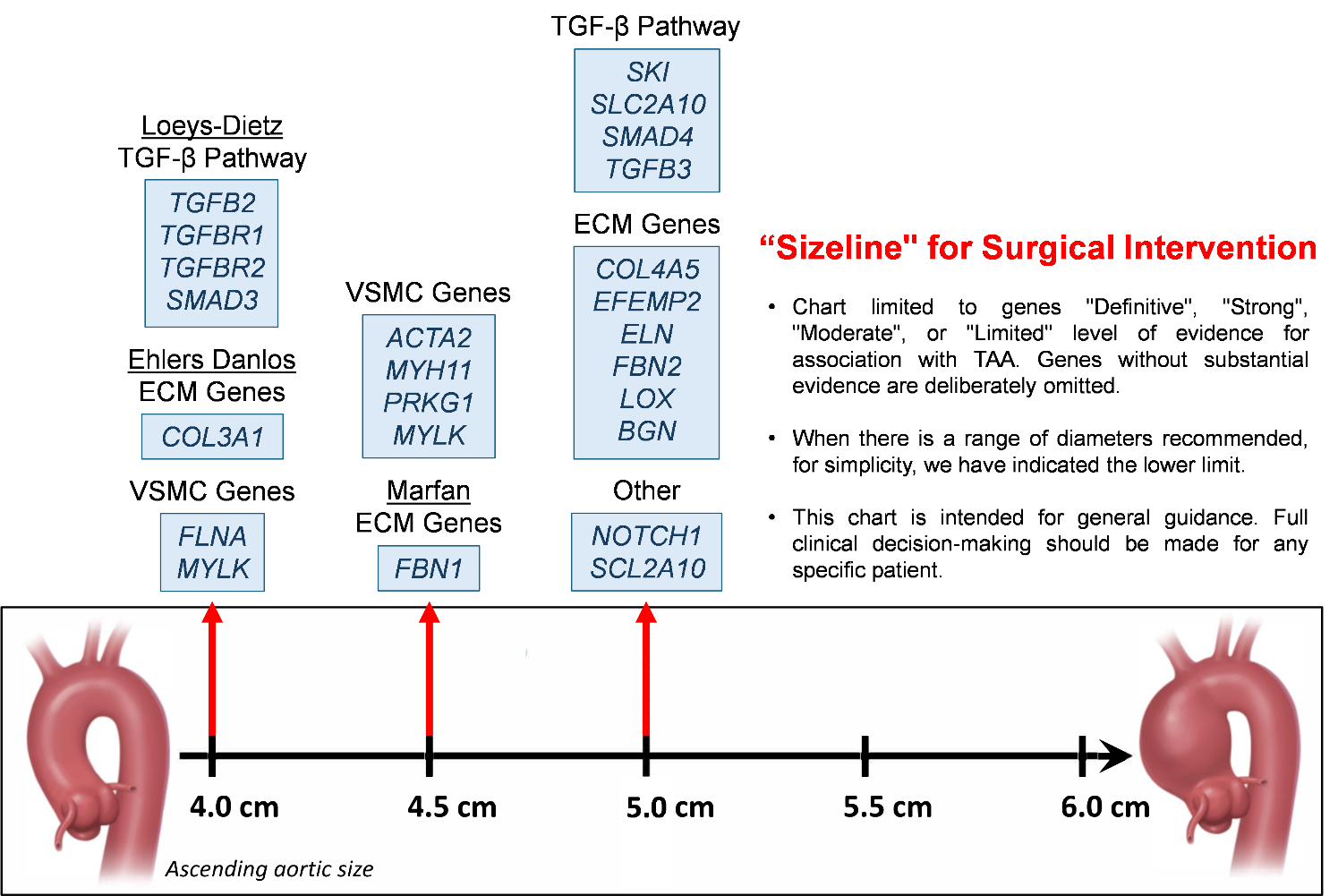


**Supplementary Figure 1.** **Gene-specific recommendations for aortic surgery.** Schematic representation of the ascending aortic diameter threshold for aortic repair surgery, based on the presence of a known pathogenic variant in a gene associated with TAAD. Image adapted from Elefteriades *et al.*, 2024 (1).

## Supplementary Tables

**Supplementary Table 1**: List of genes associated with thoracic aortic disease which have already been tested in zebrafish models. The type of genetic modification strategy is listed, as well as the targeted location and resultant allele, if applicable.

**Gene Targeting approach / mutated allele Reference**

***Morpholino knockdown***

*foxe3* translation blocking morpholino (2)

*mat2aa* not specified (3)

*skia* translation blocking morpholino (4)

*skib* translation blocking morpholino (4)

***Variant mRNA overexpression***

*acta2* G148R and R179H (5)

*smad3a* P124T, V244F, T261I, R287W, L296P and A349P (6)

***ENU mutagenesis***

*fbn2b* *te382* and *gw1* alleles (7)

*secisbp2* *sa33758* allele (8)

*smad3a* *sa2363* allele (9)

***TALEN mutagenesis***

*ltbp3* *ltbp3^fb28^*: 7 bp deletion in exon 3 (10)

***CRISPR mutagenesis (F0)***

*col1a2* crRNA for exon 4 and exon 13 (11)

*col5a1* crRNA for exon 6 and exon 7 (11)

*col5a2a* crRNA for exon 2 and exon 8 (11)

*col5a2b* crRNA for exon 2 and exon 7 (11)

*emilin1a* crRNA for exon 3 and exon 7 (11)

*emilin1b* crRNA for exon 3 and exon 7 (11)

*fbn1* crRNA for exon 2 and exon 5 (11)

*mib1* crRNA for exon 12 and exon 19 (11)

***CRISPR mutagenesis (stable line)***

*atp6v1e1b* *atp6v1e1b^cmg78^*: 1 bp deletion in exon 5 (12)

*ltbp1* *ltbp1^fb29^*: 8 bp deletion in exon 14 (10)

*ppil4* *ppil4^ya337^*: 11 bp deletion in exon 5 (13)

*robo4* *robo4^jh176^*: 7 bp deletion in exon 6 (14)

*smad3b* *smad3b^cmg10^*: 2 bp deletion in exon 3 (9)

*smad3b* *smad3b^cmg53^*: 2 bp deletion in exon 3 (9)

*smad6a* *smad6a^cmg15^*: 2 bp deletion in exon 4 (9)

*smad6a* *smad6a^cmg59^*: 14 bp insertion in exon 4 (9)

*smad6b* *smad6b^cmg13^*: 5 bp deletion in exon 1 (9)

*smad6b* *smad6b^cmg54^*: 14 bp deletion in exon 1 (9)

*tgfbr1a* *tgfbr1a^bns329^*: 4 bp del in exon 1 (15)

*tgfbr1b* *tgfbr1b^bns225^*: 8 bp del in exon 4 (15)

# Supplementary References

1. Elefteriades JA, Zafar MA, Ziganshin BA. Genetics of Aortic Aneurysm Disease: 10 Key Points for the Practitioner. *JTCVS Open* (2024) 21:58-63. Epub 20240726. doi: 10.1016/j.xjon.2024.07.014.

2. Kuang SQ, Medina-Martinez O, Guo DC, Gong L, Regalado ES, Reynolds CL, et al. Foxe3 Mutations Predispose to Thoracic Aortic Aneurysms and Dissections. *J Clin Invest* (2016) 126(3):948-61. Epub 2016/02/09. doi: 10.1172/JCI83778.

3. Guo DC, Gong L, Regalado ES, Santos-Cortez RL, Zhao R, Cai B, et al. Mat2a Mutations Predispose Individuals to Thoracic Aortic Aneurysms. *Am J Hum Genet* (2015) 96(1):170-7. Epub 20141231. doi: 10.1016/j.ajhg.2014.11.015.

4. Doyle AJ, Doyle JJ, Bessling SL, Maragh S, Lindsay ME, Schepers D, et al. Mutations in the Tgf-Beta Repressor Ski Cause Shprintzen-Goldberg Syndrome with Aortic Aneurysm. *Nat Genet* (2012) 44(11):1249-54. Epub 2012/10/02. doi: 10.1038/ng.2421.

5. Sebastian WA, Inoue M, Shimizu N, Sato R, Oguri S, Itonaga T, et al. Cardiac Manifestations of Human Acta2 Variants Recapitulated in a Zebrafish Model. *J Hum Genet* (2024) 69(3-4):133-8. Epub 20240205. doi: 10.1038/s10038-024-01221-0.

6. Sheppard MB, Smith JD, Bergmann LL, Famulski JK. Novel Smad3 Variant Identified in a Patient with Familial Aortopathy Modeled Using a Zebrafish Embryo Assay. *Front Cardiovasc Med* (2023) 10:1103784. Epub 20230228. doi: 10.3389/fcvm.2023.1103784.

7. Mellman K, Huisken J, Dinsmore C, Hoppe C, Stainier DY. Fibrillin-2b Regulates Endocardial Morphogenesis in Zebrafish. *Dev Biol* (2012) 372(1):111-9. Epub 2012/07/31. doi: 10.1016/j.ydbio.2012.07.015.

8. Schoenmakers E, Marelli F, Jorgensen HF, Visser WE, Moran C, Groeneweg S, et al. Selenoprotein Deficiency Disorder Predisposes to Aortic Aneurysm Formation. *Nat Commun* (2023) 14(1):7994. Epub 20231202. doi: 10.1038/s41467-023-43851-6.

9. Vanhooydonck M, Verlee M, Silva MS, Pottie L, Boel A, Van Impe M, et al. Early Mechanisms of Aortic Failure in a Zebrafish Model for Thoracic Aortic Dissection and Rupture. *bioRxiv* (Preprint posted online February 16, 2024). doi: 10.1101/2024.02.12.580022.

10. Abrial M, Basu S, Huang M, Butty V, Schwertner A, Jeffrey S, et al. Latent Tgfbeta-Binding Proteins 1 and 3 Protect the Larval Zebrafish Outflow Tract from Aneurysmal Dilatation. *Dis Model Mech* (2022) 15(3). Epub 20220328. doi: 10.1242/dmm.046979.

11. Prendergast A, Ziganshin BA, Papanikolaou D, Zafar MA, Nicoli S, Mukherjee S, et al. Phenotyping Zebrafish Mutant Models to Assess Candidate Genes Associated with Aortic Aneurysm. *Genes (Basel)* (2022) 13(1). Epub 20220110. doi: 10.3390/genes13010123.

12. Pottie L, Van Gool W, Vanhooydonck M, Hanisch FG, Goeminne G, Rajkovic A, et al. Loss of Zebrafish Atp6v1e1b, Encoding a Subunit of Vacuolar Atpase, Recapitulates Human Arcl Type 2c Syndrome and Identifies Multiple Pathobiological Signatures. *PLoS Genet* (2021) 17(6):e1009603. Epub 2021/06/19. doi: 10.1371/journal.pgen.1009603.

13. Barak T, Ristori E, Ercan-Sencicek AG, Miyagishima DF, Nelson-Williams C, Dong W, et al. Ppil4 Is Essential for Brain Angiogenesis and Implicated in Intracranial Aneurysms in Humans. *Nat Med* (2021) 27(12):2165-75. Epub 20211209. doi: 10.1038/s41591-021-01572-7.

14. Gould RA, Aziz H, Woods CE, Seman-Senderos MA, Sparks E, Preuss C, et al. Robo4 Variants Predispose Individuals to Bicuspid Aortic Valve and Thoracic Aortic Aneurysm. *Nat Genet* (2019) 51(1):42-50. Epub 20181119. doi: 10.1038/s41588-018-0265-y.

15. Boezio GL, Bensimon-Brito A, Piesker J, Guenther S, Helker CS, Stainier DY. Endothelial Tgf-Beta Signaling Instructs Smooth Muscle Cell Development in the Cardiac Outflow Tract. *Elife* (2020) 9. Epub 2020/09/30. doi: 10.7554/eLife.57603.
